# Negative regulation of miR‐1275 by H3K27me3 is critical for glial induction of glioblastoma cells

**DOI:** 10.1002/1878-0261.12525

**Published:** 2019-06-18

**Authors:** Jialuo Mai, Jiayu Gu, Ying Liu, Xincheng Liu, Ke Sai, Zhijie Chen, Wanjun Lu, Xiaozhi Yang, Jingyi Wang, Cui Guo, Shuxin Sun, Fan Xing, Longxiang Sheng, Bingzheng Lu, Zhu Zhu, Hongjiaqi Sun, Dongdong Xue, Yuan Lin, Jing Cai, Yaqian Tan, Chuntao Li, Wei Yin, Lin Cao, Ying Ou‐yang, Pengxin Qiu, Xingwen Su, Guangmei Yan, Jiankai Liang, Wenbo Zhu

**Affiliations:** ^1^ Department of Pharmacology, Zhongshan School of Medicine Sun Yat‐sen University Guangzhou China; ^2^ Department of Anesthesiology, Sun Yat‐Sen Memorial Hospital Sun Yat‐Sen University Guangzhou China; ^3^ Department of Infectious Disease The Third Affiliated Hospital of Sun Yat‐sen University Guangzhou China; ^4^ Department of Neurosurgery/Neuro-oncology Sun Yat-sen University Cancer Center Guangzhou China; ^5^ State Key Laboratory of Oncology in South China, Collaborative Innovation Center for Cancer Medicine Sun Yat‐sen University Cancer Center Guangzhou China; ^6^ Department of Biochemistry, Zhongshan School of Medicine Sun Yat‐sen University Guangzhou China; ^7^ Department of Pediatrics, Sun Yat‐sen Memorial Hospital Sun Yat‐sen University Guangzhou China

**Keywords:** cAMP, differentiation therapy, GBM, H3K27me3, miR‐1275

## Abstract

Activation of the cyclic adenosine monophosphate/protein kinase A (cAMP/PKA) pathway induces glial differentiation of glioblastoma (GBM) cells, but the mechanism by which microRNA (miRNA) regulate this process remains poorly understood. In this study, by performing miRNA genomics and loss‐ and gain‐of‐function assays in dibutyryl‐cAMP‐treated GBM cells, we identified a critical negative regulator, hsa‐miR‐1275, that modulates a set of genes involved in cancer progression, stem cell maintenance, and cell maturation and differentiation. Additionally, we confirmed that miR‐1275 directly and negatively regulates the protein expression of glial fibrillary acidic protein (GFAP), a marker of mature astrocytes. Of note, tri‐methyl‐histone H3 (Lys27) (H3K27me3), downstream of the PKA/polycomb repressive complex 2 (PRC2) pathway, accounts for the downregulation of miR‐1275. Furthermore, decreased miR‐1275 expression and induction of GFAP expression were also observed in dibutyryl‐cAMP‐treated primary cultured GBM cells. In a patient‐derived glioma stem cell tumor model, a cAMP elevator and an inhibitor of H3K27me3 methyltransferase inhibited tumor growth, induced differentiation, and reduced expression of miR‐1275. In summary, our study shows that epigenetic inhibition of miR‐1275 by the cAMP/PKA/PRC2/H3K27me3 pathway mediates glial induction of GBM cells, providing a new mechanism and novel targets for differentiation‐inducing therapy.

**Abbreviations**
APL: acute promyelocytic leukemia
cAMP: cyclic adenosine monophosphate
CNS: central nervous system
dbcAMP: dibutyryl‐cAMP
EdU: 5‐ethynyl‐2’‐deoxyuridine
EZH: enhancer of zeste
GBM: glioblastoma
GFAP: glial fibrillary acidic protein
GSCs: glioma stem cells
GSEA: gene senrichment analysis
H3K27me3: tri‐methyl‐histone H3 (Lys27)
miRNA: microRNA
PKA: protein kinase A
PRC2: polycomb repressive complex 2
RNA‐seq: RNA sequencing
siRNA: small interfering RNA

## 1 Introduction

Glioblastoma (GBM) is the most common and deadly cancer of the central nervous system (CNS) and is associated with a poor clinical prognosis (Wesseling and Capper, 2017). The major therapeutic strategy, which includes surgical resection, radiation, and chemical therapy, is usually applied as the standard medical management approach for patients with GBM. However, even after this standard regiment, GBM patients have a median survival of only 12–15 s (Stupp *al.*, 2005; Wen and Kesari, 2008). Although targeted molecular therapy has clear advantages in the treatment of several types of solid tumors, such as non‐small cell lung cancer, the overall impact of these advances on glioma has been disappointing (Wang *al.*, 2015). Thus, there is an urgent need for therapeutic agents and strategies that provide greater benefits.

Differentiation therapy is a strategy that involves inducing malignant cells to differentiate toward relatively benign mature cells by applying specific chemicals or signaling agents (Leszczyniecka *al.*, 2001). To date, significant progress in differentiation therapy has been made in the field of hematologic malignancies, particularly acute promyelocytic leukemia (APL). All‐trans retinoic acid and arsenic trioxide have become standard management for patients newly diagnosed with APL, and recent clinical trials reported that combination therapy using both agents resulted in complete remission rates beyond 90% (Burnett *al.*, 2015; Lo‐Coco *al.*, 2013). However, in light of the complexity of oncogenic pathways, differentiation therapy aimed at solid tumors is still under investigation. Previously, we reported that activation of the cyclic adenosine monophosphate/protein kinase A (cAMP/PKA) pathway by cholera toxin can induce malignant glioma cell differentiation into benign astrocyte‐like cells, which are characterized by proliferation arrest and glial fibrillary acidic protein (GFAP) expression (Li *al.*, 2007). Elucidating the molecular mechanisms involved in glial induction may provide valuable insights into the underlying biological features of this disease and possible new therapeutic targets.

MicroRNA (miRNA) are a large family of small non‐protein‐coding RNA of ~ 20–30 nucleotides that function by silencing RNA via perfect or imperfect base pairing with targmRNA. Through translation repression, mRNA cleavage and mRNA decay, miRNA regulate a series of biological processes, including cell differentiation, proliferation, and apoptosis. Dysregulation of miRNA is often related to human disease, especially the occurrence and progression of cancer (Lujambio and Lowe, 2012). In this study, we identified miR‐1275 as a differentiation repressor, the downregulation of which directly contributes to GFAP induction. In addition to GFAP, miR‐1275 regulates a set of genes associated with cancer progression, stem cell maintenance, cell maturation, and cell differentiation. Mechanistically, polycomb repressive complex 2 (PRC2)‐dependent tri‐methylation of histone H3 lysine 27 (H3K27me3) through cAMP/PKA pathway activation accounts for miR‐1275 downregulation. Our results suggest that decreased miRNA‐1275 expression induced by H3K27me3 drives glioma cell differentiation. These findings provide new targets for differentiation therapy.

## 2 Materials and methods

### 2.1 Cell culture and reagents

DBTRG‐05MG cells were cultured at 37 °C under 5% CO_2_ in PRIM‐1640 medium (Gibco, Thermofisher Scientific, Rockford, IL, USA) supplemented with 10% FBS (Gibco) and penicillin/streptomycin (HyClone, Thermofisher Scientific, Rockford, IL, USA). The cell line was purchased from American Type Culture Collection (ATCC, Manassas, VA, USA).

Primary patient‐derived GBM cells and glioma stem cells (GSCs) were provided by Professor Z. Chen (Department of Neurosurgery/Neuro‐oncology, Sun Yat‐sen University Cancer Center, State Key Laboratory of Oncology in South China, Collaborative Innovation Center for Cancer Medicine). Primary GBM cells were cultured in DMEM/F12 (Gibco) supplemented with 10% FBS and penicillin/streptomycin.

The following reagents were used in this study: dibutyryl‐cAMP (dbcAMP; 100 mm, dissolved in double distilled water, D0627‐1G; Sigma‐Aldrich, St. Louis, MO, USA), KT5720 (10 mm, dissolved in DMSO, B9002; APExBIO Chemicals, Houston, TX, USA), GSK J1 (10 mm, dissolved in DMSO, S7581; Selleck Chemicals,Houston, TX), and EPZ005687(10 mm, dissolved in DMSO, S7004; Selleck Chemicals, Houston, TX, USA).

### 2.2 EdU‐incorporation assay

For cell proliferation assessment, the cells were incubated with 10 mm 5‐ethynyl‐2’‐deoxyuridine (EdU; Sigma‐Aldrich) for 2 h. EdU was chemically conjugated to 50 mm Atto 488 (Sigma‐Aldrich) for 1 h. Cells were then incubated with 2.5 mg·mL^−1^ DAPI for another 30 min. Images were acquired under a fluorescence microscope (Nikon ECLIPSE Ti‐U, Tokyo, Japan).

### 2.3 Western blotting

Cells were lysed using M‐PER mammalian protein extraction reagent (Thermo Scientific, Rockford, IL, USA) or extracted total protein using ultrasonic for detecting histone protein, followed by SDS/PAGE. After being electroblotted onto a polyvinylidene fluoride membrane (Roche, Basal, Switzerland), targproteins were detected with corresponding antibodies. Antibodies targeting the following proteins were used: human GFAP (3670; Cell Signaling Technology, CST, Danvers, MA, USA), human α‐Tubulin (ARG65693; Arigo, Shanghai, China), human β‐actin (ARG62346; Arigo), human PCNA (2586; CST), human histone H3 (trimethyl K27; ab6002; Abcam, Cambridge, MA, USA), and histone H3 (9715; CST).

### 2.4 qRT‐PCR

Total RNA was extracted using TRIzol (Life Technologies, Thermo Scientific, Rockford, IL, USA) reagent and reverse‐transcribed to cDNA using a stem‐loop primer for miR‐1275 or a reverse primer for U6. Specific gene expression was quantified with SuperReal PreMix SYBR Green (TIANGEN, Beijing, China) using an Applied Biosystems 7500 fast real‐time PCR system (Life Technologies). The following amplification primers (Life Technologies) were used (50–30): miR‐1275 (stem‐loop primer, CTCAACTGGTGTCGTGGAGTCGGCAATTCAGTTGAGGACAGCCT; sense, ACACTCCAGCTCAGGTGGGGGAGAGGCTGTC; antisense, CTCAACTGGTGTCGTGGAGTCGGCAATTCAG) and U6 (sense, CTCGCTTCGGCAGCACA; antisense, AACGCTTCACGAATTTGCGT).

### 2.5 MicroRNA gain‐ and loss‐of‐function assay

Specific miRNA mimics or inhibitors were purchased from RiboBio (Guangzhou, China). Cells were transfected with miRNA using Lipofectamine RNAiMAX (13778150; Thermo Fisher) in OPTI‐MEM (31985‐070; Thermo Fisher) following the manufacturer’s instructions. Briefly, cells were seeded in 35‐mm dishes at 70–80% confluence and then transfected with 25 μg miRNA mimics or inhibitors with Lipofectamine RNAiMAX under serum‐reduced condition. After 24 h, culture media were replaced with a serum‐containing medium, and cells were treated with drugs. RNA and proteins were extracted after 72 h postdrug treated.

### 2.6 Transcriptome data processing and analysis

Raw reads were trimmed to 90 nucleotides (nt) of length and were mapped to the UBSC human genome browser database (UCSC) hg19 reference genome (Lander al., 2001) (http://genome.ucsc.edu/) using tophat (v2.0.11) (Trapnell al., 2009) (http://tophat.cbcb.umd.edu/). Default Tophat settings were used, except for the redefined parameter ‘–mate‐inner‐dist 200’. The inner distance between pair ends was estimated by the picard program (http://picard.sourceforge.net). The HTSeq program was used to count mapped reads with the parameters ‘‐s no –a 20’ (Anders al., 2015). Genes with less than two reads per million were removed, and 13 333 genes were included for further analysis. Gene count normalization and differential expression analysis were performed using the deseq package (Anders and Huber, 2010).

### 2.7 Gene annotation and functional enrichment analysis

The piano package (Varemo al., 2013) was used for gene senrichment analysis (GSEA) (Subramanian al., 2005). Gene sets were retrieved from Gene Ontology (http://geneontology.org/), Kyoto Encyclopedia of Genes and Genomes, and the Ingenuity Pathways database (IPA; Ingenuity Systems, http://www.ingenuity.com) (Kanehisa and Goto, 2000; Kanehisa al., 2014). Raw *P* values from the GSEA were corrected for multiple testing by false discovery rate. Pathways with corrected *P* values of < 0.05 were considered significant.

### 2.8 Dual‐luciferase reporter assay

Firefly and Renilla luciferase reporter plasmids were purchased from RiboBio. The fragments were amplified by PCR with primers for pmir‐GFAP‐3′‐UTR, (sense) GCGGCTCGAGACCCAGCAACTCCAACTAA and (antisense) AATGCGGCCGCCCCCAGGTGGCAGGACGTC. miRNA targsite mutants were generated using the following primer: GCGGCTCGAGACCCAGCAACTCCAACTAACAAGAAACTCAGGGGGTTGGGGCAGTCTGGAGGGGC.

Luciferase reporter assays were conducted by cotransfecting DBTRG‐05MG cells with miRNA fragments and the firefly and Renilla luciferase plasmids using lipofectamine 3000 (L3000015; Thermo Fisher). At 48 h post‐transfection, cells were harvested, and the luciferase activity was measured using a Dual‐Glo Reporter Assay System (E2920; Promega, Madison, WI, USA). Luciferase activity was calculated as the ratio of firefly luciferase activity (reporter) to Renilla luciferase activity (control).

### 2.9 RNAi experiments

Specific small interfering RNA (siRNA) targeting PKA was purchased from RiboBio. siRNA were transfected using Lipofectamine RNAiMAX (Life Technologies) with OPTI‐MEM (Life Technologies) following the manufacturer’s instructions.

### 2.10 ChIP

ChIP assays were performed based on the protocol from the manufacturer (17‐10086; Merck Millipore, Sigma‐Aldrich, St. Louis, MO, USA). Briefly, cells (1 × 10^7^) were treated with 1% formaldehyde for 10 min to cross‐link the histones to the DNA. After sonication of cell pellets, the lysate was incubated with 10 μL of anti‐K27 tri‐methylated histone H3 (ab6002; Abcam). To collect the immunoprecipitated complexes, magnetic beads were added and incubated with the lysate overnight at 4 °C. After the cross‐linking was reversed, DNA was extracted and purified using the phenol/chloroform method, ethanol‐precipitated, and dissolved in water. ChIP products were assayed via SYBR Green ChIP‐qPCR using the following set of primers: (sense) GCAGAAATACCTCACCAAGTTTTTA and (antisense) TTTGGCATACTTACAGACACAAGAC, encompassing the pri‐miR‐1275 promoter region.

### 2.11 Assessing histone methyltransferase activity

Cells were treated with the indicated compounds for 24 h. Then, according to the instructions provided in, nuclear extracts were prepared using EpiQuik™ Nuclear Extraction Kit (OP‐0002; Epigentek, Farmingdale, NY, USA). We used an EpiQuik™ histone methyltransferase activity/inhibition assay kit (P‐3005; Epigentek) to perform histone methyltransferase activity assays based on the manufacturer’s protocol. The resulting absorbance was measured at 450 nm using a Synergy H1 microplate reader (BioTek, Winooski, VT, USA).

### 2.12 Subcutaneous xenograft GBM model

Animal experiments were approved by the animal care ethics committees at Zhongshan School of Medicine, Sun Yat‐sen University. Mice were housed in a pathogen‐free animal facility. The hindflanks of 4‐week‐old female BALB/c‐nu/nu mice were subcutaneously inoculated with 1 × 10^5^ GSCs. After 7 days, palpable tumors developed (50 mm^3^), and the mice were randomly divided into three groups (*n* = 5). These four groups were injected intraperitoneally once per day with vehicle, 20 mg·kg^−1^ luteolin, 100 mg·kg^−1^ GSK J4, or combination of luteolin and GSK J4, respectively, for 14 days. Tumor diameters were measured every other day with a caliper, and tumor volumes were estimated using the following formula: width^2 ^× length/2 = *V* (mm^3^). Tumors were dissected and fixed for immunohistochemical and *in situ* hybridization (ISH) analysis. Data are means ± standard deviation (SD) of 5 mice/group.

### 2.13 Statistical analysis

Graphed results are expressed as the means ± SD, as indicated in the figure legends, for the indicated number of observations. The data were analyzed with one‐way ANOVA or Student’s *t*‐test (two‐tailed, unequal variance).

## 3 Results

### 3.1 MicroRNA expression profile in DBTRG‐05MG cells under exposure to dibutyryl‐cAMP

**Figure 1:**
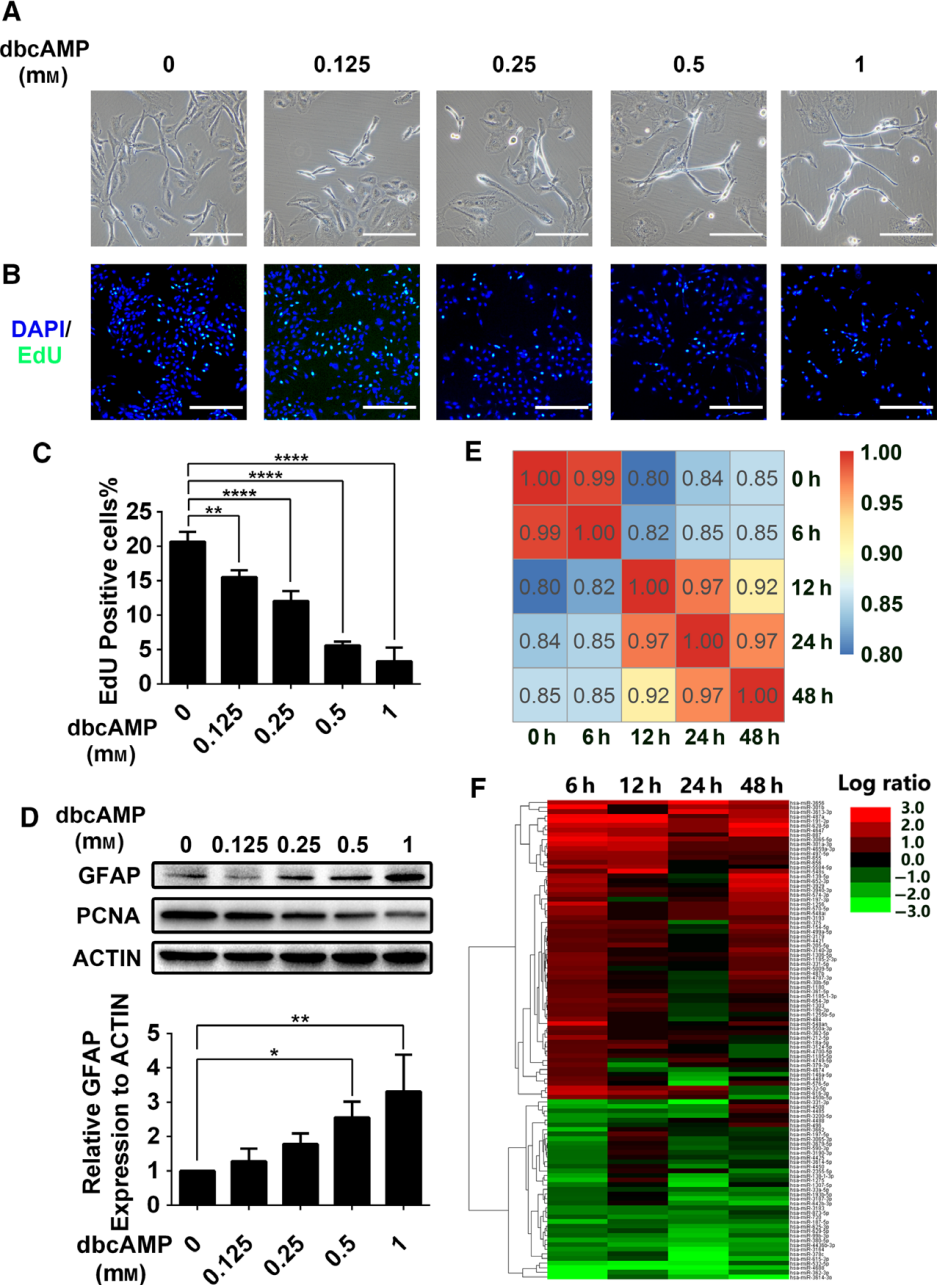
miRNA expression profile in DBTRG‐05MG cells exposed to dbcAMP. (A) Dose effects of dbcAMP on morphology changes in DBTRG‐05MG cells. Cells were treated with 0.125–1 mm dbcAMP for 3 days, and then, phase‐contrast microscopy images were captured. Scale bar, 200 μm. (B) Dose effects of dbcAMP on DBTRG‐05MG cell proliferation rate. Cells were treated with 0.125 mm to 1 mm dbcAMP for 3 days. EdU‐incorporation assays were performed at the endpoint, and then, fluorescence images were captured. EdU‐positive cells are shown in green. DAPI is shown in blue. Scale bar, 500 μm. (C) Histogram of EdU‐positive cells treated with different doses of dbcAMP (*n* = 3). Error bars indicate SD. Statistical analysis was performed with one‐way ANOVA. **, *P* < 0.01; ****, *P* < 0.0001. (D) Western blot analysis of GFAP and the proliferation marker PCNA in DBTRG‐05MG cells treated with different doses of dbcAMP for 3 days. β‐Actin was used as the loading control (upper). Histogram of relative GFAP expression in cells treated with dbcAMP (lower, *n* = 3). Error bars indicate SD. Statistical analysis was performed with one‐way ANOVA. *, *P* < 0.05; **, *P* < 0.01. (E) Correlation analysis of DBTRG‐05MG cells treated with 1 mm dbcAMP at different time points. (F) Heatmap of the miRNA expression levels in dbcAMP‐treated DBTRG‐05MG cells.

As we reported previously, activation of the cAMP signal pathway by cholera toxin or cAMP activator can induce differentiation of rat glioma cells and human glioma cells toward an astrocyte type (He *al.*, 2011; Li *al.*, 2007). dbcAMP, a cAMP analog, was reported to be used to trigger glial induction (Adornetto *al.*, 2013; Hu *al.*, 2008; Xing *al.*, 2017), and we used it as a differentiation inducer in our research. In contrast with the mainly flattened and polygonal shape of the control cells, the morphology of the dbcAMP‐treated DBTRG‐05MG cells resembled that of mature neural cells, with smaller, round cell bodies and longer processes (Fig. 1A). Next, a EdU incorporation assay was performed on dbcAMP‐treated glioma cells to evaluate their proliferation capacity. As shown in Fig. 1B,C, the average percentage of EdU‐positive cells in the control group was 22%, while the percentage in the dbcAMP‐treated group was as low as 5%. Furthermore, dbcAMP induced DBTRG‐05MG cells to express GFAP, the marker of mature astrocytes (Fig. 1D). Taken together, these data suggest that treatment with dbcAMP can induce GBM cell transformation into an astrocyte‐like phenotype, manifested by morphological changes, suppression of proliferation, and induction of GFAP expression.

Next, we conducted transcriptome and miRNA genomics analyses to further exploring genetic and epigenetic mechanisms underlying chemically induced glial differentiation. From the transcriptome data, we observed clear and dramatic changes in gene expression patterns at 12 h under dbcAMP treatment (Fig. 1E). Of note, the miRNA expression profile underwent a marked change at 6 h (Fig. 1F), earlier than the change in gene expression. Thus, to determine whether miRNA participate in glial induction via gene regulation, we identified miRNA with a fold change > 3 at 6 h and 12 h. As presented in Tables 1 and 2, 34upregulated and 18 downregulated miRNA were considered screening candidates.

**Table 1:** miRNA expression levels increased more than three‐fold in dbcAMP‐treated DBTRG cells.

miRNA	6 h	12 h
hsa‐miR‐3656	**4.21232123**	**4.86468647**
hsa‐miR‐487a	**7.37183718**	**10.9449945**
hsa‐miR‐191‐3p	**10.5324533**	**8.51265127**
hsa‐miR‐628‐5p	**5.26622662**	**4.86468647**
hsa‐miR‐139‐5p	**3.45993086**	1.38969202
hsa‐miR‐301b	**8.42574257**	1.21562156
hsa‐miR‐3065‐5p	**7.37183718**	**6.08030803**
hsa‐miR‐3613‐3p	**3.15951595**	1.21562156
hsa‐miR‐652‐3p	**3.92470753**	1.10538946
hsa‐miR‐887	**5.79262926**	**3.64851485**
hsa‐miR‐3929	**3.15951595**	1.21562156
hsa‐miR‐4647	**4.21232123**	**3.6479648**
hsa‐miR‐301a‐3p	**5.79262926**	**4.86468647**
hsa‐miR‐1256	**5.26622662**	1.21562156
hsa‐miR‐4659a‐3p	**4.21232123**	**4.86468647**
hsa‐miR‐3940‐3p	**3.68518009**	1.82375584
hsa‐miR‐497‐5p	**3.42205114**	**3.3434149**
hsa‐miR‐655	**3.2641883**	**3.28288605**
hsa‐miR‐570‐5p	**3.15951595**	1.21562156
hsa‐miR‐548ai	**3.15951595**	1.21562156
hsa‐miR‐212‐5p	**3.36955565**	1.21579411
hsa‐miR‐5584‐5p	**4.21232123**	**3.6479648**
hsa‐miR‐548an	**6.3190319**	1.21562156
hsa‐miR‐32‐5p	**4.73927393**	2.43234323
hsa‐miR‐616‐3p	**5.26622662**	**3.64851485**
hsa‐miR‐450b‐5p	**3.00864236**	2.08453803
hsa‐miR‐4525	1.05280528	**6.08030803**
hsa‐miR‐656	2.4563783	**3.24193548**
hsa‐miR‐766‐3p	1.05335534	**3.64851485**
hsa‐miR‐660‐3p	1.05280528	**3.6479648**
hsa‐miR‐431‐3p	1.57975798	**5.47249725**
hsa‐miR‐3129‐3p	1.05280528	**6.08030803**
hsa‐miR‐548s	2.10671067	**7.2970297**
hsa‐miR‐624‐5p	1.05335534	**4.25632563**

The significance items have been highlighted in Bold fonts.

**Table 2:** miRNA expression levels decreased by more than 70% in dbcAMP‐treated DBTRG cells.

miRNA	6 h	12 h
hsa‐miR‐4508	**0.26802784**	**0.22107347**
hsa‐miR‐331‐3p	**0.22563467**	0.42183677
hsa‐miR‐3662	**0.21051474**	1.21579411
hsa‐miR‐3065‐3p	**0.26312895**	1.51965906
hsa‐miR‐138‐1‐3p	**0.26312895**	0.91174045
hsa‐miR‐380‐5p	**0.26312895**	0.30382183
hsa‐miR‐187‐5p	**0.21051474**	0.48636164
hsa‐miR‐532‐5p	**0.17549365**	**0.13779779**
hsa‐miR‐1275	**0.21051474**	1.70215574
hsa‐miR‐4688	**0.10525737**	**0.12153542**
hsa‐miR‐362‐3p	**0.08096447**	0.37411168
hsa‐miR‐3614‐3p	**0.08096447**	0.46759729
hsa‐miR‐324‐5p	1.10836469	**0.19195369**
hsa‐miR‐3648	1.05297892	**0.20256645**
hsa‐miR‐24‐1‐5p	0.63176419	**0.24307083**
hsa‐miR‐4436b‐3p	0.42113946	**0.24318082**
hsa‐miR‐769‐3p	1.05301364	**0.24307083**
hsa‐miR‐490‐3p	1.26352838	**0.24318082**

The significance items have been highlighted in Bold fonts.

### 3.2 Identification of miR‐1275 as a key regulator that contributes to proliferation inhibition and differentiation induction

**Figure 2:**
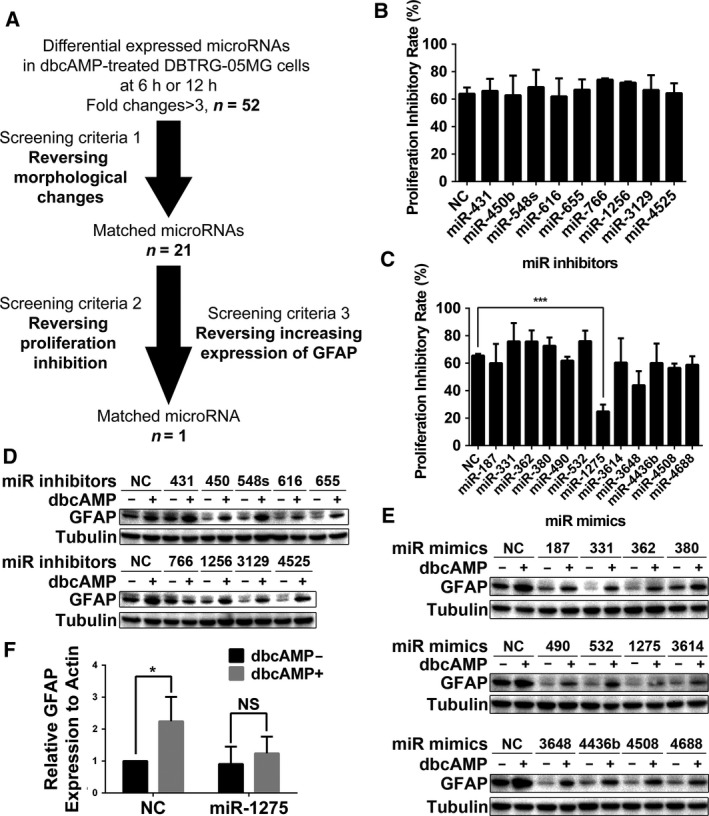
Identification of miR‐1275 as a key regulator that contributes to proliferation inhibition and differentiation induction. (A) Schematic diagrams of the miRNA found to be critical in dbcAMP‐induced GBM cell differentiation (left) and miRNA gain‐ and loss‐of‐function assays (right). (B) Quantification of proliferation inhibition rate of dbcAMP‐treated DBTRG‐05MG cells transfected with miRNA inhibitors (*n* = 3). Error bars indicate SD. (C) Quantification of the proliferation inhibition rate of dbcAMP‐treated DBTRG‐05MG cells transfected with miRNA mimics (*n* = 3). Error bars indicate SD. Statistical analysis was performed with one‐way ANOVA. ***, *P* < 0.001. (D) Western blot analysis of GFAP in dbcAMP‐treated DBTRG‐05MG cells transfected with miRNA inhibitors. Tubulin was used as the loading control. (E) Western blot analysis of GFAP in dbcAMP‐treated DBTRG‐05MG cells transfected with miRNA mimics. Tubulin was used as the loading control. (F) Quantification of western blot analysis of GFAP in dbcAMP‐treated DBTRG‐05MG cells transfected with miR‐1275 mimics (*n* = 3). Error bars indicate SD. Statistical analysis was performed with Student’s *t*‐test. *, *P* < 0.05.

To identify the differentially expressed miRNA (DEmiRs) critical for dbcAMP‐induced differentiation, we performed gain‐ or loss‐of‐function assays for 52 miRNA candidates in DBTRG‐05MG cells (Fig. 2A). In this screening assay, we assessed the contributions of every miRNA to differentiation induction by evaluating the effects of miRNA mimics or inhibitors on morphology, proliferation, and GFAP expression. Based on the screening strategy above, we first identified 21 of 52 DEmiRs whose mimics or inhibitors reversed the morphology alteration induced by dbcAMP. Through a second screening cycle, we finally identified miR‐1275, whose mimic successfully decreased both the proliferation inhibition and GFAP expression (Fig. 2B–E and Fig. S1A,B) caused by dbcAMP. We repeated the gain of miR‐1275 function assay for three times (Fig. S1C) and concluded that miR‐1275 mimics statistically abrogated increased expression of GFAP by dbcAMP (Fig. 2F). These data strongly indicate that miR‐1275 plays a critical role in dbcAMP‐induced glioma differentiation.

### 3.3 miR‐1275 negatively regulates a set of genes involved in cell development and cell maturation

**Figure 3:**
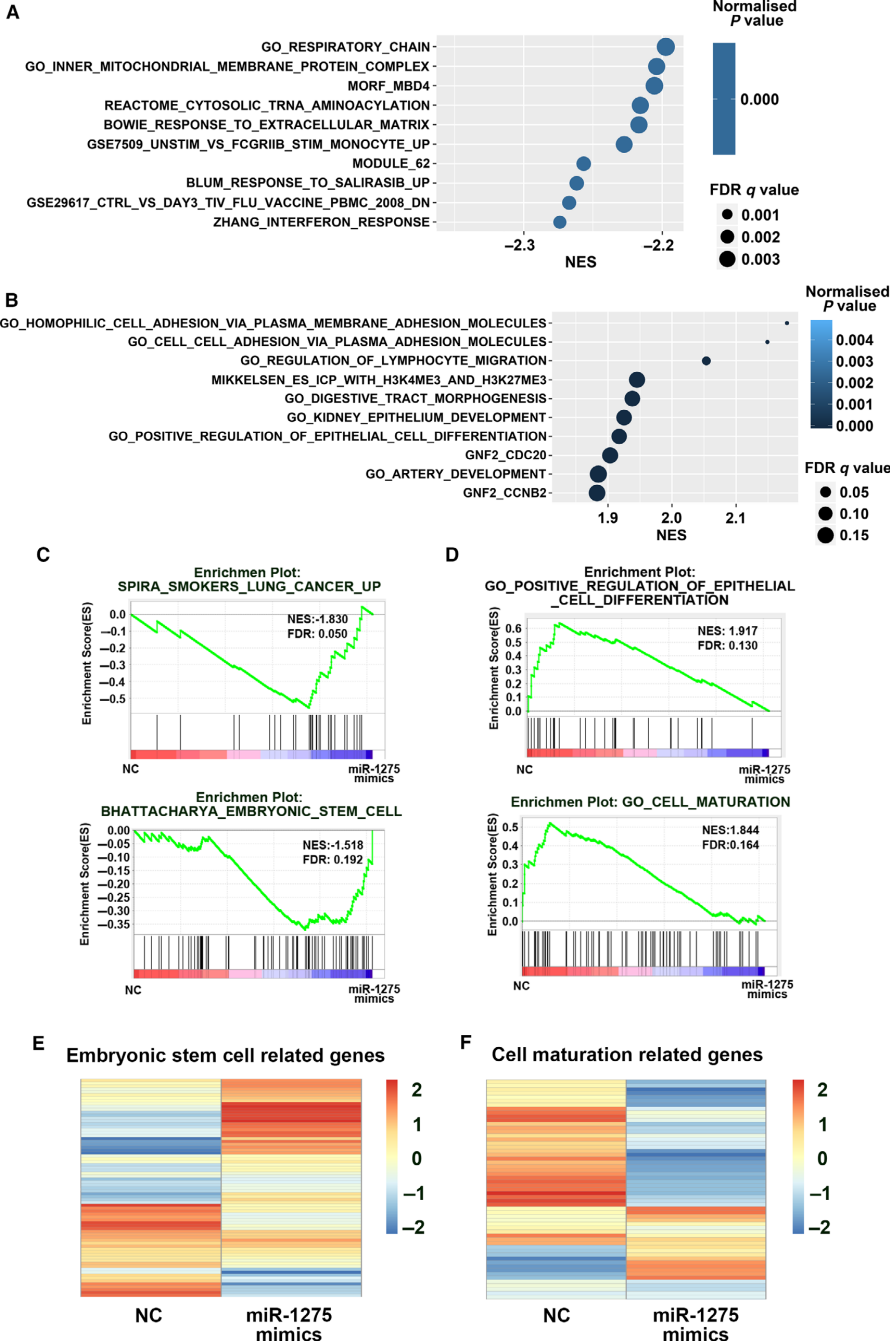
miR‐1275 negatively regulates a set of genes involved in cell development and cell maturation. (A) GSEA of transcriptome data performed by comparing DBTRG‐05MG cells transfected with miR‐1275 mimic with control cells. The top 10 most enriched upregulated pathways are listed. Normalized enrichment scores are shown in the bar chart. (B) GSEA of transcriptome data performed by comparing DBTRG‐05MG cells transfected with miR‐1275 mimic with the negative control cells. The top 10 most enriched downregulated pathways are listed. Normalized enrichment scores are shown in the bar chart. (C) GSEA enrichment plots of genes ranked based on miR‐1275 mimic‐treated versus SPIRA_SMOKERS_LUNG_CANCER_UP and BHATTACHARYA_EMBRYONIC_STEM_CELL gene sets. (D) GSEA enrichment plots of genes ranked based on miRNA mimic NC‐treated versus GO_POSITIVE_REGULATION_OF_ EPITHELIAL_CELL_DIFFERENTIATION and GO_CELL_MATURATION gene sets. (E) Heatmap of the expression level of embryonic stem cell genes. High expression is shown in red, and low expression is shown in blue. (F) Heatmap of the expression level of cell maturation. High expression is shown in red, and low expression is shown in blue.

To elucidate the regulatory network of miR‐1275, we conducted a transcriptome assay of DBTRG‐05MG cells transduced with miR‐1275 mimic using RNA sequencing (RNA‐seq) experiments. By performing GSEA of the transcriptome data, we determined the top 10 upregulated and downregulated pathways. As shown in Fig. 3A, mitochondrial and immunological related genes were upregulated in the miR‐1275 mimic group. As expected, several gene sets related to tissue morphogenesis, development, and cell differentiation were downregulated in miR‐1275‐transduced GBM cells (Fig. 3B). By analyzing enrichment plots, we observed that miR‐1275 significantly upregulated cancer‐related and embryonic stem cell‐related gene sets (Fig. 3C) but downregulated gene sets related to cell type differentiation and maturation (Fig. 3D). The heatmaps, showing the expression profile of embryonic stem cell‐related (Fig. 3E) and cell maturation‐related genes (Fig. 3F), further confirmed the inhibitory role of miR‐1275 in GBM cell differentiation.

### 3.4 Negative contribution of miR‐1275 to GFAP expression through direct targeting of mRNA

The regulatory molecule responsible for the GFAP expression upregulation in glial induction of GBM cells induced by cAMP signal activators has not been identified. In this study, we obtained evidence of an inverse correlation between miR‐1275 and GFAP expression. Therefore, we sought to further confirm whether miR‐1275 directly regulates GFAP expression.

**Figure 4:**
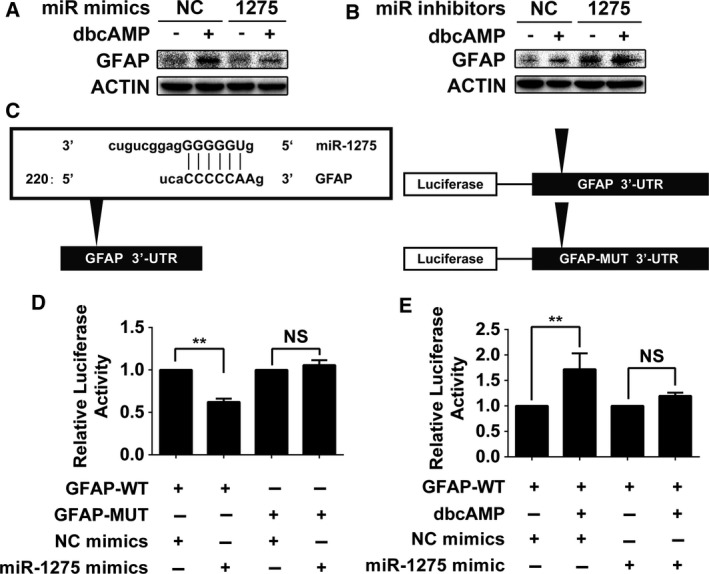
Negative contribution of miR‐1275 to GFAP expression by targeting the 3′‐UTR. (A) Western blot analysis of GFAP in dbcAMP‐treated DBTRG‐05MG cells transfected with miR‐1275 mimic. β‐Actin was used as the loading control. (B) Western blot analysis of GFAP in dbcAMP‐treated DBTRG‐05MG cells transfected with miR‐1275 inhibitor. β‐Actin was used as the loading control. (C) Left panel: the predicted binding site of miR‐1275 in the GFAP 3′‐UTR is indicated (arrowhead). The complementary sequences between GFAP and miR‐1275 are indicated above the arrowheads. The nucleotide position of the targsite is indicated relative to the position of the GFAP stop codon (the first nucleotide after the stop codon of GFAP is defined as 1). Right panel: schemes for the reporter vectors; the wild‐type GFAP 3′‐UTR sequence (upper) and mutant GFAP 3′‐UTR sequence (lower) are shown. Black triangles indicate predicted miR‐1275 binding sites within the 3′‐UTR of the GFAP gene. (D) Luciferase reporter assays with the GFAP‐3′‐UTR reporter vector and miRNA fragments in DBTRG‐05MG cells. The luciferase values are relative to the Renilla luciferase activity (*n* = 3). Error bars indicate SD. Statistical analysis was performed with one‐way ANOVA. **, *P* < 0.01. NS, not significant. (E) Luciferase reporter assays with the wild‐type GFAP‐3’‐UTR reporter vector and miRNA fragments in dbcAMP‐treated DBTRG‐05MG cells. The luciferase values are relative to the Renilla luciferase activity (*n* = 3). Statistical analysis was performed with one‐way ANOVA. Error bars indicate SD. **, *P* < 0.01. NS, not significant.

As shown in Fig. 4A,B, a miR‐1275 mimic reversed GFAP expression induced by dbcAMP, while a miR‐1275 inhibitor upregulated expression of GFAP similar to dbcAMP. Furthermore, through sequence blasting, we determined that the 3′‐UTR of GFAP contains a predicted binding site for miR‐1275. To validate the regulatory activity of miR‐1275 toward GFAP, we constructed luciferase plasmids with wild‐type or mutant GFAP 3’‐UTR sequences and transferred them into DBTRG‐05MG cells together with the miR‐1275 mimic (Fig. 4C). As shown in Fig. 4D, the miR‐1275 mimic reduced the luciferase activity of DBTRG‐05MG cells containing the wild‐type luciferase plasmid by 40% while having no impact on cells containing the mutant plasmid. In addition, dbcAMP enhanced the luciferase signal, which was abolished by the miR‐1275 mimic (Fig. 4E). Taken together, these data suggest that GFAP is a direct targof miR‐1275.

### 3.5 H3K27me3 downstream of PKA/PRC2 signaling mediates miR‐1275 downregulation

**Figure 5:**
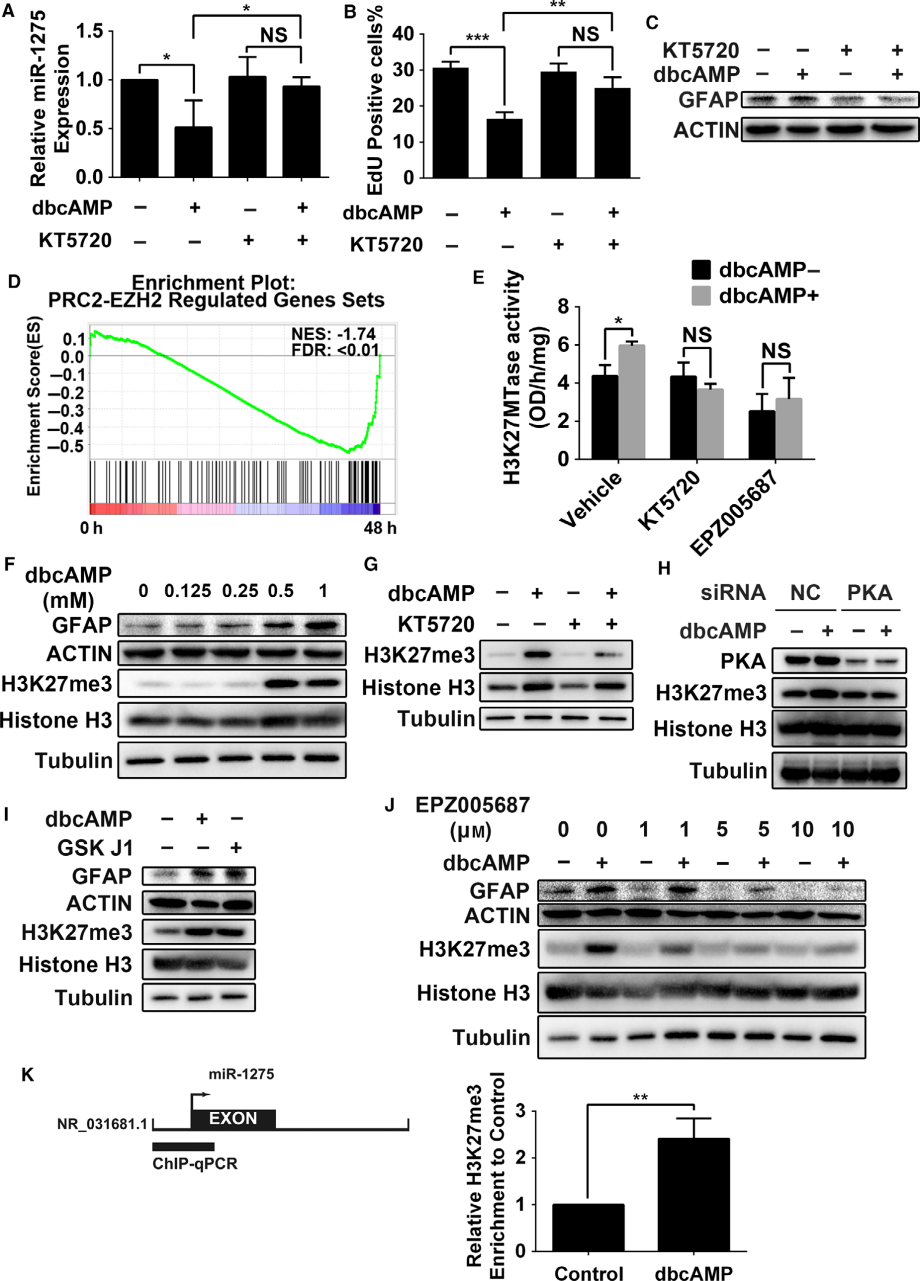
PRC2‐dependent H3K27me3 downstream of PKA mediates miR‐1275 downregulation. (A) The effects of KT5720 on miR‐1275 expression in dbcAMP‐treated DBTRG‐05MG cells (*n* = 3). Error bars indicate SD. Statistical analysis was performed with one‐way ANOVA. *, *P* < 0.05. NS, not significant. (B) The effects of the PKA inhibitor KT5720 on the proliferation rate of dbcAMP‐treated DBTRG‐05MG cells (*n* = 3). Error bars indicate SD. Statistical analysis was performed with one‐way ANOVA. **, *P* < 0.01; ***, *P* < 0.001. NS, not significant. (C) Western blot analysis of the effects of KT5720 on GFAP expression in dbcAMP‐treated DBTRG‐05MG cells. β‐actin was used as loading control. (D) GSEA enrichment plot of genes ranked based on dbcAMP‐induced differentiation versus the PRC2_EZH2_DN.V1_DN gene set. (E) Quantification of H3K27MTase activity in DBTRG‐05MG cells treated with 0.5 mm dbcAMP (*n* = 3), 10 μm KT5720, and 10 μm EPZ005687 for 3 days. Error bars indicate SD. Statistical analysis was performed with one‐way ANOVA. *, *P* < 0.05. NS, not significant. (F) Western blot analysis of GFAP and H3K27me3 in DBTRG‐05MG cells treated with different doses of dbcAMP for 3 days. β‐Actin and histone H3 were used as the loading controls. (G) Western blot analysis of H3K27me3 in DBTRG‐05MG cells treated with 0.5 mm dbcAMP and 10 μm KT5720 for 3 days. Histone H3 was used as the loading controls. (H) Western blot analysis of PKA and H3K27me3 in DBTRG‐05MG cells transfected with negative control siRNA (siNC) or siPKA and then treated with 0.5 mm dbcAMP for 3 days. Tubulin and histone H3 were used as the loading controls. (I) Western blot analysis of GFAP and H3K27me3 in DBTRG‐05MG cells treated with 0.5 mm dbcAMP or 10 μm GSK J1 for 3 days. β‐Actin and histone H3 were used as the loading controls. (J) Western blot analysis of GFAP and H3K27me3 in DBTRG‐05MG cells treated with 0.5 mm dbcAMP and different doses of EPZ005687 for 3 days. β‐Actin and histone H3 were used as the loading controls. (K) A diagrammatic representation of the pri‐miR‐1275 promoter is shown (left panel). The transcription start site (arrow) and location of pri‐miR‐1275 (black box) are indicated. Thick bars represent the region analyzed by ChIP‐PCR. The H3K27me3 status in the pri‐miR‐1275 promoter region before and after dbcAMP exposure is shown (right panel, *n* = 3). Statistical analysis was performed with Student’s *t*‐test. Error bars indicate SD. **, *P* < 0.01.

Protein kinase A is one of the typical kinases activated by cAMP, and thus, we used the PKA inhibitor KT5720 to test whether suppression of miR‐1275 expression is mediated by PKA activation. As shown in Fig. 5A–C, the expected decrease in both miR‐1275 expression and the number of EdU‐positive cells, as well as the expected increase in GFAP expression, was abolished by KT5720 in dbcAMP‐treated DBTRG‐05MG cells.

To gain further mechanistic insight into signals downstream of PKA, we performed GSEA focusing on changes in epigenetic‐related proteins from Molecular Signatures Database. Surprisingly, upregulation of the PRC2_EZH2_DN.V1_DN gene swas observed during the dbcAMP‐induced differentiation of DBTRG‐05MG cells, indicating that the PRC2 signal pathway was activated (Fig. 5D). PRC2, a kind of polycomb repressive complex containing the enzymatic subunit enhancer of zeste 1 (EZH1) or EZH2, has histone methyltransferase activity and primarily trimethylates histone H3 on lysine 27 (H3K27me3) (Boyer *al.*, 2006; Cao *al.*, 2002). To gain further insight into the relation between the cAMP/PKA pathway and PRC2, we measured the enzymatic activity of H3K27 methyltransferase (H3K27MTase) during exposure to dbcAMP, KT5720 inhibiting PKA, or EPZ005687 inhibiting EZH1/2. The results showed that dbcAMP increased the activity of H3K27MTase and that inhibiting either PKA or EZH1/2 abrogated the H3K27MTase activation induced by dbcAMP (Fig. 5E), suggesting that PRC2 activation is mediated by PKA.

Next, we tested the expression level of H3K27me3 in dbcAMP‐treated DBTRG‐05MG cells, and significant upregulation was observed (Fig. 5F). Moreover, dbcAMP‐induced upregulation of H3K27me3 was abolished by either of PKA‐specific inhibitor KT5720 or knocking down PKA with specific siRNA fragments (Fig. 5G,H). Furthermore, to clarify whether enhanced H3K27me3 contributes to glial induction of GBM cells, we applied the selective H3K27me3 histone demethylase inhibitor GSK J1 and the selective EZH1/2 inhibitor EPZ005687. GSK J1 induced increases in H3K27me3 and GFAP, in agreement with the data showing the effects of dbcAMP (Fig. 5I). Conversely, EPZ005687 abolished the increase in GFAP expression induced by dbcAMP (Fig. 5J). These results indicate that activation of the PKA/PRC2/H3K27me3 signaling pathway mediates glial induction of DBTRG‐05MG cells.

To further clarify whether miR‐1275 expression is directly regulated by PRC2 ‐dependent H3K27me3, we performed a ChIP‐qPCR assay. In response to dbcAMP treatment, enrichment of repressive H3K27me3 marks in the promoter region of pri‐miR‐1275 was increased more than twofold compared with the control, indicating that miR‐1275 transcription is suppressed through an epigenetic mechanism in dbcAMP‐treated GBM cells (Fig. 5K).

Taken together, these data indicate that cAMP/PKA signaling activates PRC2, which in turn increases H3K27me3 and decreases miR‐1275 expression.

### 3.6 miR‐1275 downregulation is involved in dbcAMP‐induced differentiation in human GBM cells derived patient and xenografts derived from GSCs

**Figure 6:**
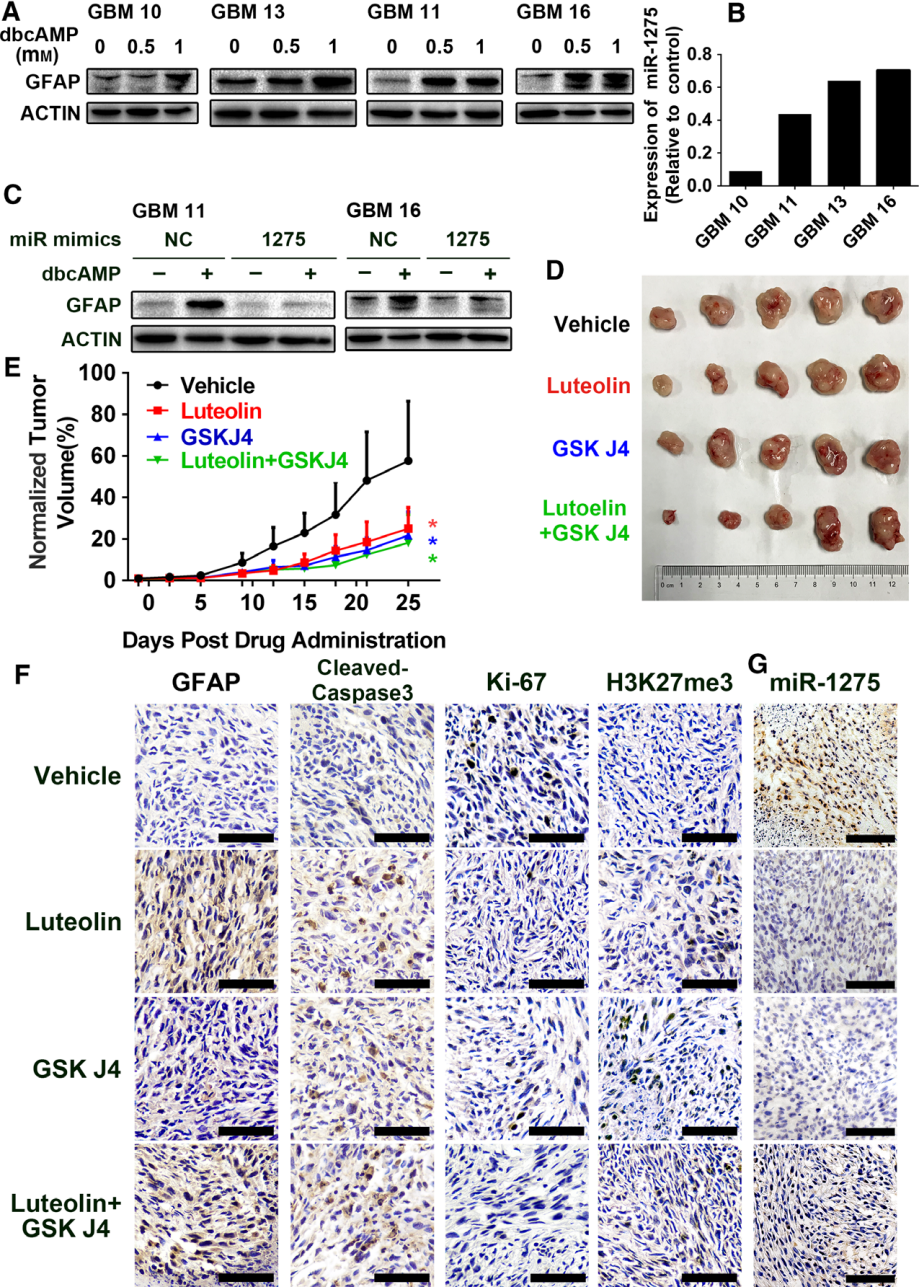
miR‐1275 downregulation is involved in dbcAMP‐induced differentiation in human GBM cells derived patient and xenografts derived from GSCs. (A) Effects of dbcAMP on GFAP expression in primary cultured human GBM cells. Cells were treated with 1 mm dbcAMP for 3 days, and β‐actin was used as the loading control. (B) Effects of dbcAMP on miR‐1275 expression in primary cultured human GBM cells. Cells were treated with 1 mm dbcAMP for 3 days. (C) Western blot analysis of GFAP in dbcAMP‐treated primary cultured human GBM cells transfected with miR‐1275 mimic. β‐Actin was used as the loading control. (D) Nu/nu mice were subcutaneously inoculated with patient‐derived GSCs (*n* = 5 per group). Seven days after inoculation, mice were intraperitoneally treated with vehicle, luteolin (20 mg·kg^−1^·day^−1^), GSK J4 (100 mg·kg^−1^·day^−1^), or combination of luteolin and GSK J4; 30 days after inoculation, the tumor‐bearing mice were sacrificed and tumor from each group was photographed. (E) The tumor volume (means ± SDs) was recorded every other day after inoculation. Statistical analysis was performed with repeated‐measure ANOVA. *, *P* < 0.05. (F) Tumor from the mice were subjected to immunohistochemistry to evaluate the expression of GFAP, cleaved caspase 3, Ki‐67, and H3K27me3. Scale bar, 100 μm. (G) ISH was performed on tumor from mice xenograft model to evaluate expression of miR‐1275. Scale bar, 100 μm.

To further confirm the clinical potential of cAMP activators as drug candidates and miR‐1275 as a targfor therapy‐induced differentiation of human GBM, we tested the effects of dbcAMP on differentiation and examined the expression of miR‐1275 in primary cultured human glioma cells. As shown in Fig. 6A, dbcAMP prompted primary GBM cells to produce more GFAP, indicating a pro‐differentiation effect of cAMP signaling activation in primary cultures from four different clinical glioma tissues. Meanwhile, quantitative RT‐PCR data revealed that dbcAMP reduced miR‐1275 expression (Fig. 6B). Moreover, transfection of primary cultured glioma cells with the miR‐1275 mimic prevented dbcAMP‐induced upregulation of GFAP (Fig. 6C). Thus, these results suggest a pivotal role for miR‐1275 in cAMP enhancer‐induced primary GBM cell differentiation.

Next, to evaluate the antitumor and pro‐differentiation efficacy of cAMP elevator luteolin targeting PDE and H3K27me3 elevator GSK J4 targeting H3K27 methyltransferase, we engineered a subcutaneously xenografts in nude mice using patient‐derived GSCs. In tumor‐bearing mice, we intraperitoneally administrated luteolin which elevates endogenous cAMP level via inhibiting phosphodiesterase with a high liposolubility and bioactivity in a dose of 20 mg·kg^−1^ once per day, and/or GSK J4 which is the prodrug of GSK J1 in a dose of 100 mg·kg^−1^ once per day. As indicated by the tumor growth curves and tumor mass indices, luteolin alone, GSK J4 alone, and the combination suppressed tumor growth equally (Fig 6D,E). In addition, treatment with luteolin and/or GSK J4 decreased cell counts with Ki‐67‐positive signal and increased signals of GFAP, H3K27me3, and cleaved caspase 3 in GSCs xenografts (Fig. 6F). Moreover, reduced expression of miR‐1275 was also observed in either group (Fig. 6G). Taken together, our data revealed that luteolin elevating cAMP signals and GSK J4 elevating H3K27me3 levels have clinical potential in treating GBM by downregulating expression of miR‐1275.

## 4 Discussion

We found that miR‐1275 transcriptionally constrains the expression of the mature astrocyte marker GFAP and is negatively regulated by cAMP/PKA/PRC2/H3K27me3 signaling. Intriguingly, downregulation of miR‐1275 accounts for the glial induction of GBM cells. Our findings suggest that miR‐1275, downstream of histone methylation, functions as a pivotal regulator for maintaining the malignant phenotype of GBM cells.

MicroRNA are important epigenetic regulatory effectors that regulate gene expression in many biological processes by blocking translation of specific mRNA, and their dysregulation has been found to participate in a series of cancers (Stefani and Slack, 2008). In our study, we found that dbcAMP‐induced GBM differentiation led to decreased miR‐1275 expression, which suppressed GFAP expression by binding its 3′‐UTR, revealing a new epigenetic regulation mechanism for GFAP. Moreover, transcriptomics data revealed that miR‐1275 regulates a set of genes involved in cell development, maturation, and histone methylation. Our data strongly support the idea that miR‐1275 is closely related to the dedifferentiated state of GBM cells and may function as an onco‐miRNA in the carcinogenesis process. Intriguingly, in the GSCs differentiation process, repression of miR‐1275 has been found to cause increased expression of the oligodendroglial lineage marker CLDN11 (Katsushima *al.*, 2012). In two different cell contexts, GBM cells, and GSCs, miR‐1275 plays a negative role in cell differentiation induction by blocking the expression of maturation‐related genes; thus, its loss is leads to phenotype transformation.

In addition to genetic aberrations, abnormalities in epigenetic regulators, including the histone methyltransferase PRC2 and its substrate H3K27me3, also contribute to GBM. EZH2, enzymatic subunit of PRC2, has been reported to be overexpressed in GBM (Li *al.*, 2013; Suva *al.*, 2009) and is often correlated with metastasis and poor prognosis (Chase and Cross, 2011; Simon and Lange, 2008), suggesting that EZH2 may act as an oncogene, and that targeting EZH2 can be a potential therapeutic approach for pediatric gliomas (Mohammad *al.*, 2017). Interestingly, loss‐of‐function EZH2 mutations can also promote cancer in a context‐dependent manner (Morin *al.*, 2010; Simon *al.*, 2012) and prolonged inhibition of EZH2 in GBM causes a cell fate switch toward a more undifferentiated and proliferative state, which results in tumor progression(de Vries *al.*, 2015). Moreover, recent studies have suggested that the H3K27 methylation (H3K27M) mutation plays an important role in the pathogenesis of GBM (Khuong‐Quang *al.*, 2012; Schwartzentruber *al.*, 2012; Wu *al.*, 2012). H3K27me3 is widely believed to be associated with gene silencing (Boyer *al.*, 2006; Lee *al.*, 2006; Roh *al.*, 2006). In our study, GBM cells exposed to the treatment drug displayed an increase in PRC2 activity and elevation of H3K27me3, mediating subsequent downregulation of miR‐1275 and glial differentiation via epigenetic mechanism. All these results strongly indicate an important role of PRC2/H3K27M epigenetic regulation in GBM.

Protein kinase A can phosphorylate a large number of substrates in the cytoplasm and nucleus (Shabb, 2001). Recent studies have reported that activation of cAMP‐dependent PKA initiates mesenchymal‐to‐epithelial transformation by phosphorylating PHF2, a histone demethylase for histone H3 at lysine 9 (H3K9) (Pattabiraman *al.*, 2016). In our study, we identified another histone methyltransferase, PRC2 containing EZH1/2, as a PKA substrate. These results indicate that PKA positively modulates the activities of multiple epigenetic enzymes, which provides a rational explanation for the differentiation‐inducing property of the cAMP/PKA signal activator.

Glioblastoma is one of the deadliest and most refractory cancer types among cancers of the CNS, and therapeutic strategies aimed at prolonging overall survival are under current investigation. Our data suggest that the cAMP/PKA pathway is a promising targpathway for GBM therapeutics (Li *al.*, 2007; Shu *al.*, 2012). Activation of cAMP signaling can decrease glioma cell proliferation, induce glial transformation of glioma cells, and inhibit the growth of brain tumor xenografts (Goldhoff *al.*, 2008; Yang *al.*, 2007). In this study, we further clarified that miR‐1275 inhibitors cause proliferation inhibition and glial differentiation in GBM cells. Importantly, we found that glioma‐bearing nude mice treated with Luteolin and GSK J4, the prodrug of GSK J1, manifested slower tumor growth relative to mice treated with vehicle. These data were in strong agreement with the data, showing that JMJD3 inhibition to increase H3K27 methylation by applying GSK J4 exhibited potent antitumor activity against pediatric brainstem glioma (Hashizume *al.*, 2014). These results provided laboratory evidence that the application of cAMP activators, miR‐1275 antagonists, and H3K27me3 enhancers may be beneficial in differentiation therapy for malignant glioma. Thus, the cAMP/PKA/PRC2/H3K27me3 pathway has promising potential for the development of differentiation‐inducing therapeutic targets for GBM.

## 5 Conclusion

In summary, we found that the expression of the mature astrocyte marker GFAP is transcriptionally suppressed by miR‐1275, which is negatively regulated by cAMP/PKA/PRC2/H3K27me3 signaling. Moreover, downregulation of miR‐1275 contributes to the glial induction of GBM cells. Our results showed that epigenetic inhibition of miR‐1275 by the cAMP/PKA/PRC2/H3K27me3 pathway mediates glial induction of GBM cells.

## Conflict of interest

The authors declare no conflict of interest.

## Author contributions

JM, JG, and YLiu designed and performed the experiments and analyzed the data. ZC performed experiments of culture of primary patient‐derived GBM cells and GSCs. XL performed the transcriptome and proteome data analysis. WZ and JL conceived the study and participated in the experimental design and in the analysis and interpretation of data. KS, XY, WL, JW, CG, SS, FX, LS, BL, ZZ, HS, DX, YLin, JC, YT, CL, WY, LC, YO, PQ, XS, and GY contributed to data acquisition, interpretation, and data assembly. JM, WZ, and JL edited the manuscript. JM, JG, and YLiu contributed equally to this work.

## Supporting information


**Fig. S1.** Verification of miR‐1275 as a key regulator that contributes to differentiation induction. (A) Western blot analysis of GFAP in dbcAMP‐treated DBTRG‐05MG cells transfected with miRNA inhibitors. Tubulin was used as the loading control. (B) Western blot analysis of GFAP in dbcAMP‐treated DBTRG‐05MG cells transfected with miRNA mimics. Tubulin was used as the loading control. (C) Western blot analysis of GFAP in dbcAMP‐treated DBTRG‐05MG cells transfected with miR‐1275 mimic. β‐Actin was used as the loading control.

## Data Availability

The accession number for the raw read data of RNA‐seq reported in this paper is GEO: GSE89745 and GSE119161.
