# Supplementary figures and images for: Negative regulation of miR‐1275 by H3K27me3 is critical for glial induction of glioblastoma cells
Source: Mol Oncol. 2019 Jun 18;13(7):1589–604. doi: 10.1002/1878-0261.12525 (PMC6599839; doi:10.1002/1878-0261.12525)

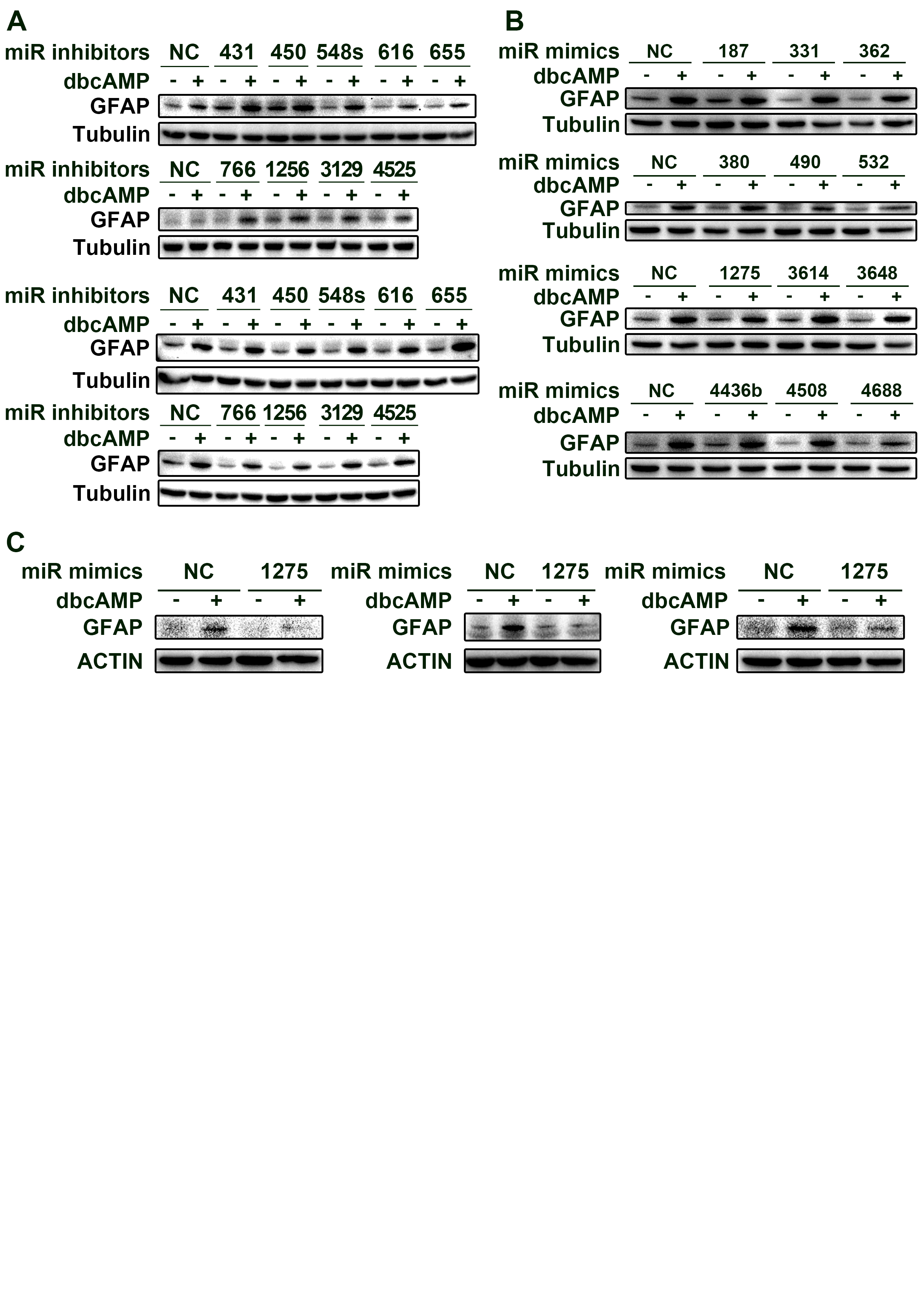

Supplement: Supplementary file 1 — Fig. S1. Verification of miR‐1275 as a key regulator that contributes to differentiation induction. (A) Western blot analysis of GFAP in dbcAMP‐treated DBTRG‐05MG cells transfected with miRNA inhibitors. Tubulin was used as the loading control. (B) Western blot analysis of GFAP in dbcAMP‐treated DBTRG‐05MG cells transfected with miRNA mimics. Tubulin was used as the loading control. (C) Western blot analysis of GFAP in dbcAMP‐treated DBTRG‐05MG cells transfected with miR‐1275 mimic. β‐Actin was used as the loading control. [file MOL2-13-1589-s001.tif]
